# Flow Control in Porous Media: From Numerical Analysis to Quantitative μPAD for Ionic Strength Measurements

**DOI:** 10.3390/s21103328

**Published:** 2021-05-11

**Authors:** Pouya Mehrdel, Hamid Khosravi, Shadi Karimi, Joan Antoni López Martínez, Jasmina Casals-Terré

**Affiliations:** 1 Mechanical Engineering Department—Microtech Lab., Universitat Politecnica de Catalunya, C/Colom 7-11, CP 08222 Terrassa, Spain; hamid.khosravi@upc.edu (H.K.); shadi.karimi@upc.edu (S.K.); jasmina.casals@upc.edu (J.C.-T.); 2 Department of Mining, Industrial and ICT Engineering (EMIT), Universitat Politecnica de Catalunya, AV. Bases de Manresa 61-73, CP 08240 Manresa, Spain; joan.antoni.lopez@upc.edu

**Keywords:** microfluidic paper-based analytical devices, colorimetric detection, quantitative assay, numerical simulation, computational fluid dynamics, ionic strength, diffusion assay

## Abstract

Microfluidic paper-based analytical devices (µPADs) are a promising technology to enable accurate and quantitative in situ assays. Paper’s inherent hydrophilicity drives the fluids without the need for external pressure sources. However, controlling the flow in the porous medium has remained a challenge. This study addresses this problem from the nature of the paper substrate and its design. A computational fluid dynamic model has been developed, which couples the characteristics of the porous media (fiber length, fiber diameter and porosity) to the fluidic performance of the diffusion-based µPAD sensor. The numerical results showed that for a given porous membrane, the diffusion, and therefore the sensor performance is affected not only by the substrate nature but also by the inlets’ orientation. Given a porous substrate, the optimum performance is achieved by the lowest inlets’ angle. A diffusion-based self-referencing colorimetric sensor was built and validated according to the design. The device is able to quantify the hydronium concentration in wines by comparison to 0.1–1.0 M tartaric acid solutions with a 41.3 mM limit of detection. This research showed that by proper adjustments even the simplest µPADs can be used in quantitative assays for agri-food applications.

## 1. Introduction

In the past decades, microfluidic technology has proven its capabilities to be used in the chemical, biochemical, food, agri-food, pharmaceutical and medical fields [1,2,3,4]. Their fast-responsiveness, low reagent requirement, accuracy and user-friendliness persuade both the researchers and entrepreneurs to focus more on this technology and expand its applicability to new fields and, specifically into the field of Micro Total Analysis Systems (µTAS) [5,6,7,8]. Although the microfluidic platforms are extremely efficient, they mostly rely on the utilization of external energy sources to control the flow, and they require the incorporation of sophisticated detection techniques. Therefore, in situ measurements or the use in less developed regions is seriously limited. Researchers have tried to address this last issue by taking advantage of the generated capillary flow in the porous media, which introduced the microfluidic paper-based analytical devices (µPAD) [9,10,11,12]. However, the capillary flow is linked to its complex control, which is related to the paper characteristics. This fact restricts the µPADs applications mainly to qualitative detection if they are not supported by sophisticated detection techniques.

Porous media are tangled matrices of fibers (normally cellulosic) that provide an ideal environment for functionalization. However, their intrinsic variability is a challenge for microfluidic designers, and most current successful devices are qualitative or yes/no assays. Some researchers have taken advantage of the fiber matrix to trap nanoparticles and enhance the surface-to-volume ratio [13,14,15,16]. This fact has increased its reliability and repeatability. However, the use of µPADs (by non-trained personnel) as quantitative assays is still in development. For instance, the most conventional and well-known µPADs applications are paper-based pH indicators and pregnancy tests (hCG; human Chorionic Gonadotropin hormone test) [9]. Both use markers (either pH indicator or antibody) immobilized within the porous media. The interaction within the understudy solution and the coated marker causes the expected color change. Nevertheless, there are reports of developed paper-based sensors that can be used in diverse applications by taking advantage of interdisciplinary methods. Examples of such application are reported in the literature, for instance in electrochemical (EC) [17,18], chemiluminescence (CL) [14,18,19] and electrochemiluminescence (ECL) [18,20] methods to quantify different compounds. These quantification methods increase the assays’ accuracy and widen the applicability horizon. However, the use of the aforementioned methods does not help to simplify the tests and conflicts with the idea of using µPADs in less developed regions.

Even though, there are numerous reports of developed quantitative µPAD platforms based on laborious detection techniques. There are also remarkable µPADs that provide quantitative or semi-quantitative measurements without the need to use such complex methods. For instance, Kim et al. [21] developed a quantitative colorimetric assay for the detection of Tropinin I, which enhanced the results due to the use of gold nanoparticles. A similar approach was used by Parolo et al. [22] that took advantage of Lateral Flow Immunoassay (LFIA) and used gold nanoparticles to capture human IgG and presented the results through colorimetric techniques. Walczak et al. (2009) [23] proposed a colorimetric assay capable of quantifying down to 5 ng/mL of cocaine in the samples. Gerold et al. [24] managed to distinguish 1.0–2.5 mM of potassium ion on a paper-based microfluidic device by taking advantage of the selective distance-based quantification method. Additionally, there have been reports about the simultaneous detection of several compounds [25,26] or employing smart-phones for the evaluation of the assays [27,28]. However, in most of the aforementioned studies, the research relied on the attachment of the analyte to the pre-treated particles/detectors/substrates, and the effects of the substrate on the devices themselves were not considered.

Other than the requirement of pre-treated substrates or particles, certain assays, such as enzyme-linked immunosorbent assay or ELISA, require a sequential flow of the reagents to the detection zone. Since the fluid movement is not controllable in a porous media, careful strategies should be considered to achieve the sequential arrival of the reagents to the reaction zones. The strategies to control the flow can be categorized into three types: geometrical-based, chemical-based and mechanical-based [29,30]. The geometrical-based methods rely on the change of the channel length, the channel width and any obstruction of the flow path that can delay the fluid arrival to the detection zone. Apilux et al. [31] managed to perform an automatic ELISA for hCG (human Chorionic Gonadotropin hormone) determination by using a baffled paper-based microfluidic channel. Another geometrical-based flow control method is based on the successive placement of fluid sources on the channel to create a paper network. The fluid from the closest source arrives faster than the other sources, and this enables a programmed delivery of reagents to the detection zone. Fu et al. [32] took advantage of this method and introduced a µPAD, which detected malaria proteins in a sandwich immunoassay. Geometrical-based flow control can also be achieved by using shunts. As the fluid is absorbed by a fluid shunt, the flow is slowed down. Once the shunt is filled, the flow gains its initial velocity [29]. Chemical-based flow control relies on altering the porous characteristics of the substrate when compounds are deposited in the voids (pores). For instance, Lutz et al. [33] deposited different amounts of dissolvable sugar to the paper substrate and showed that the fluid flow could be delayed significantly. The mechanical-based flow control is extremely useful for three-dimensional µPADs. This method enables the possibility to place mechanical valves, which create connections between paper-substrates at different levels. In summary, the abovementioned strategies are ideal for sequential flow assays. However, they do not focus on applications, where co-laminar flows are required, and they do not study the effect of porous media characteristics and geometry on the fluids’ interaction.

In one of the well-studied but rare researches, Osborn et al. [34] investigated the effect of the source position in a T-mixer and its influence on the flows within the porous medium. The study displayed that if one of the sources (in the inlets) is placed closer to the inlets’ intersection, the respective flow would become dominant in the main channel and overcome the flow from the other inlet. The study pointed out the complexity of controlling the flow in porous media. However, the need for manual positioning and therefore the flow synchronization would decrease the accuracy of this type of device if used in assays.

This study aims to investigate the influence of paper properties and geometry on the flow characteristics. Specifically, the influence on the diffusion in paper-based microfluidic platforms. First, we have modeled the flow behavior in a given porous media, by taking into account the porous media characteristics (fiber length, fiber diameter, porosity) and also the geometry of the porous media microfluidic circuit. Later, based on the outcome, we have conceptualized a self-referencing sensor with a straightforward 3D-printed structure to guarantee the simultaneous contact between the reagents and paper strip that could be utilized everywhere and characterized its accuracy and sensitivity.

The novel proposed sensor can measure the sample’s unknown ionic concentration (wine) by comparing its diffusion width to the diffusion width of a solution with a known ionic concentration (tartaric acid). The diffusion width is measured using colorimetric analysis. The optimization of the porous media substrate avoids the use of pre-treated substrates/particles and more sophisticated detection techniques and brings quantification capacity to a paper-based sensor.

## 2. Methodology

In this section, we present an overview of the governing equations of the flow in porous media. We describe the model of the different studied geometries as well as the materials and experimental approach.

### 2.1. Sensor Description

The colorimetric sensor measures the diffusion width of hydronium ions [H_3_O^+^]. The main goal is to monitor the diffusion width through the color change across the measurement line and compare it to the diffusion width of a known sample (self-referencing approach).

The use of the proposed sensor as a threshold sensor or a comparative sensor to a given known ionic strength solution, brought the need to use three inlets: one for the unknown solution, one for the known ionic strength solution and a third solution as a mediator or label (in this study pH indicator). The pH indicator transforms the ionic concentration into a colorimetric signal and enables a simultaneous comparison between the diffusion widths of both solutions (with known and unknown ionic strengths).

**Figure 1:**
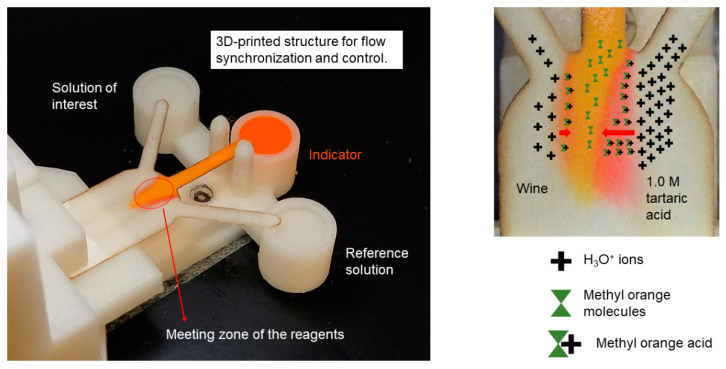
Picture of the ionic strength wine evaluation using a diffusion-based µPAD and the flow control system. Red arrows illustrate the diffusion widths due to unequal ionic concentration of solutions in the meeting zone.

With respect to the abovementioned points, the proposed sensor has three inlets: the left one for the sample of interest, the middle one for indicator and the right one for the reference sample. In order to achieve a proper performance of the sensor, the device uses a 3D-printed support, see Figure 1. This support and inlets’ geometry have been designed to guarantee that the inlets of the paper substrate contact the fluid reservoirs at the same time and the fluids flow within the porous medium, simultaneously. Therefore, the solutions encounter at the main channel (meeting zone) when required, and the chemical reaction can be monitored. When the H_3_O^+^ ions of the solution diffuse into the pH indicator, they show a certain color change. If the ionic strength is higher, there will be more ions and therefore more diffusion. If the ionic concentration of the reference solution is known, then the ionic concentration of the solution of interest can be found by comparing its diffusion width to the reference one, as it is shown in Figure 1.

### 2.2. Principles of Numerical Simulation

#### 2.2.1. Fluid Flow and Mixing Phenomena

Computational fluid dynamics (CFD) is a derivation of the finite volume method that discretizes the solving domain into smaller cells and solves the Navier–-Stokes equations in each cell. Ansys Fluent CFD software is chosen for solving the equations of the fluid flow through the porous media substrate. We assumed that the fluid is incompressible, Newtonian and the process is isothermal.

For solving the Navier–-Stokes equation, a coupled scheme is chosen for the pressure-velocity coupling. Due to the dimensions, laminar flow is the governing regime and the energy and thermal sources are negligible, the continuity (1) and momentum (2) equations solved at each cell are:

(1) $$\nabla \vec{U}=0,$$

(2) $$\rho \vec{U}\cdot \nabla \vec{U}=-\nabla P+\mu \nabla^{2}C$$

In the above equations $\vec{U}$ is the velocity vector, ρ is the density of the working fluid, ∇P is the pressure gradient and C is the species concentration within the solving domain.

A user defined scalar is introduced to model the diffusion of species in the control volume.

The assumption of laminar flow implies that the convection-diffusion Equation (3) describes the transport phenomena in the domain of interest. The convection-diffusion equation is defined as:

(3) $$\rho \vec{U}\cdot \nabla C=D\nabla^{2}C,$$

Let the *D* be the diffusion coefficient of the species.

In order to simulate the diffusion in the problem, the Equation (3) is solved simultaneously with continuity and momentum Equations (1) and (2) at each iteration.

#### 2.2.2. Numerical Modelling of the Paper Substrate

The substrate is defined as a porous medium and the geometry is segregated into three zones. Two zones are the lateral inlet branches and the other zone contains the middle inlet and main channel. The porous medium is defined in Fluent by its porosity and the viscous resistance values (1/permeability (α)), which are identical for all the zones. The only difference is in their magnitude of the X and Y vectors. These values are calculated based upon the inlets’ angle and defined in the opposite direction of the flow to define the resistance against the fluid movement.

It is worthy to mention that the inertial resistance is neglected in the model, since it is completely overshadowed by the viscous resistance. Calculations showed that the viscous resistance is greater than the inertial resistance by approximately 1 × 10^3^ magnitude of order. Also, it should be noted that the authors are aware of the fact that the wetting phenomenon swells the fibers. But since the material of the paper-substrate is the same in all the cases and the same reagents are used, the authors have presumed that the permeability and the viscous resistance has remained unchanged for the sake of simplification.

#### 2.2.3. Porous Medium

Whatman grade 5 paper, bought from @Fisher Scientific, is used as the assay substrate.

Porous media are tortuous matrices of fibers that are packed together creating some voids (or in other words; pores) [35]. The fluid flows within the pores and is affected by the size of the pores and their distribution. One of the most important characteristics of the porous medium is its porosity (*ε*) that can be calculated through:

(4) ε=VvVO

where, $V_{v}$ and $V_{O}$ are representing the void volume and the body volume of the understudy medium.

The total volume of the paper consists of the voids volume plus the fibers (or particles) volume. Therefore, the porosity of the medium can be correlated with the density of the whole porous medium ($\rho_{0}$) and the density of its constituent fibers/particles ($\rho_{p}$). As a result, the porosity can be calculated as:

(5) ε=1−ρpρ0

The permeability (*α*) of the porous medium, according to Yazdchi et al. [36], can be obtained from the physical and geometrical properties of the substrate, rather than the pressure drop and flow velocity within the porous medium. The equation is described as:

(6) $$\frac{\alpha }{d^{2}}=\frac{\varepsilon^{3}}{\varphi {\left(\frac{L_{e}}{L_{f}}\right)}^{2}{\left(1-\varepsilon \right)}^{2}}$$

where, $L_{f}$, *L*, *d* and *φ* are fiber’s length, substrate’s total length, fiber’s diameter and the pore shape factor, respectively.

Table 1 summarizes the geometrical and physical properties of cellulose fibers, present in Whatman 5 paper. Therefore, from Equation (5), the Whatman 5 paper porosity can be obtained (0.6467). The permeability (α) is calculated from equation 6, being 4.551 × 10^−15^ m^2^, which was then used in the definition of porous zone in the Fluent Model.

Porous medium also affects the effective diffusion coefficient. The geometry of the substrate will be optimized based on the comparison of the diffusion of the user-defined scalar in the numerical models. According to Giri [37], the diffusion coefficient of a dye molecule (mathematically defined as the scalar) is reported to be 2 × 10^−10^ m^2^/s. Ansys Fluent software requires the diffusion coefficient (D) to be in [kg/m.s] unit. Therefore, D is multiplied by the fluid density before using it in computations. Furthermore, to model the porous zone in the Fluent Model, the effective diffusion coefficient (*D_eff_*) is calculated through [38]:

(7) $$D_{eff}=\left(D\cdot \rho \right)\cdot \varepsilon$$

Therefore, according to Equation (7) the effective diffusion coefficient in the Fluent model is set to 1.29 × 10^−7^ kg/m·s.

#### 2.2.4. Boundary Conditions

The initial scalar’s concentration is assumed to be 0.05 M in lateral channels. Besides, the working fluid is assumed to be liquid water at RT (25 °C) and 50% (HR), see Table 1. The initial flow velocity at the inlets is set to 1.39 × 10^−4^ m/s (value estimated from initial measurements of the time required to fill the inlet branches). The convergence residuals are set to 1 × 10^−10^ for all the criteria. Other properties, such as the pressure outlet condition, the density of fluid, the diffusivity of the scalar, the porosity and the viscous resistance (1/permeability) are summarized in Table 2.

#### 2.2.5. Grid Independency

**Figure 2:**
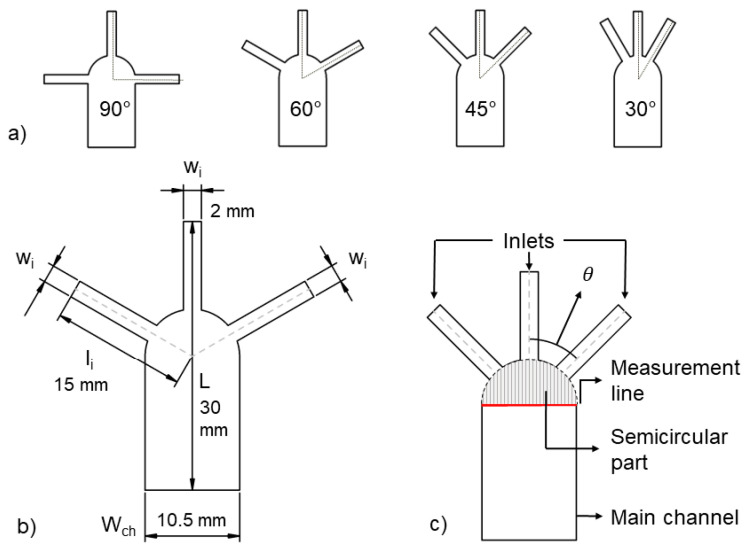
Schematics of the substrate geometry. (**a**) from left to right: Substrate geometries of the 90, 60, 45 and 30-degree models. Channel width (W_ch_), paper strip length (L), inlets branch width (w_i_) and measurement line are also displayed in (**b**,**c**).

A grid independency analysis is done to make sure that the size of the discretized cells does not affect the final results. Six mesh densities of the 90-degree model (see Figure 2) are compared.

The boundary conditions and the solving methods are identical in all the cases.

The minimum element size of the mesh schemes varies from 500 μm in the “Low” mesh density to 25 μm in the “Ultra-fine” mesh density (detailed description of the mesh schemes is presented in the Appendix A). Due to the porous nature of the control surface, the differences in the velocity profiles are quite insignificant. As a result, the scalar distribution in the different mesh designs is set as a reference for comparison.

#### 2.2.6. Diffusion Evaluation Methods

One of the most common methods to evaluate the distribution of species is the comparison of the scalar concentrations at each cell to the median value [39]. This method uses the following dimensionless and normalized ratio: (σ).

(8) $$\sigma =\frac{\left(C_{i}-\bar{C}\right)}{\left(C_{max}-\bar{C}\right)}$$

where *C_i_* is the calculated concentration at the *i*th cell. $\bar{C}$ denotes the median concentration in the solving domain and *C_max_* is the highest concentration of species. By obtaining the normalized ratio *σ*, the mixing quality (*M.Q*) is calculated in each mesh scheme.

(9) $$M.Q=1-\sqrt{\frac{1}{N}\times \overset{N}{\underset{1}{\sum }}\left(\sigma^{2}\right)}$$

where *N* is the number of discretized cells in each mesh density and it depends on the minimum element size. The larger the minimum element size, the smaller the number of cells.

### 2.3. Geometry

Figure 2a shows the four different models created to study the effect of the inlets’ orientation on the diffusion width. The angle between the middle inlet and the lateral inlets varies from 90°, 60°, 45° and 30° degrees. Hereinafter, the introduced geometries are referred to as 90-degree, 60-degree, 45-degree and 30-degree models.

For a proper comparison, all the models share the same total length of the paper strip, the required fluid volume to fill the inlet’s branch and the distance from the fluid source to the center of the main channel. Therefore, the total length (L), inlets’ width (w) and the channel width (W_ch_) are set to 30 mm, 2 mm and 10.5 mm, respectively. (Please see Figure 2b)

The top of the main channel starts with a semi-circular shape, see Figure 2c. This decision is made in order to make sure that the change in inlets’ angle does not affect other characteristics of the design. The semi-circular part has a 10.5-mm diameter and its center is exactly located at the middle of the main channel. Figure 1 and Figure 2c show that the addition of this part not only guarantees the equal geometrical preconditions for all the models, but also brings flexibility in designing different models.

The length of the inlets’ branches (l_c_ = l_i_ − W_ch_/2) from the fluid source up to the edge of the semi-circular part is kept constant due to the presence of this circular beginning of the main channel. Therefore, l_c_ is 9.75 mm in all the models, regardless of the inlets’ angle.

The measurement line, as shown in Figure 2c, is set at the intersection of the main channel and the semi-circular’s part.

### 2.4. Experimental Setup

In order to verify the numerical simulation results and to evaluate the proposed sensor performance, several experimental assays are designed. The studied geometries (30-degree, 45-degree and 90-degree models) are laser cut (using NEJE7000mW laser) in Whatman grade 5 paper.

A 3D-printed support is developed to keep the paper substrate fixed. This printed support facilitates a simultaneous contact of the three inlets of the paper strip to the reagents.

To evaluate the results, pictures are taken at different time intervals and the diffusion widths are studied over the measurement line. It should be noted that to avoid viscosity variations all the experiments are conducted at the room temperature (RT, 25 °C).

#### 2.4.1. Reagents

Conventional white table wine (pH 3.25 at RT) is supplied locally. Tartaric acid (2,3-Dihydroxybutanedioic acid), is bought from @Merck Schuchardt OHG. Tartaric acid is one of the main components of wines. Three concentrations of 0.1, 0.5 and 1.0 M of tartaric acid are prepared. Measurements at the RT shows that the prepared tartaric acid solution had a pH of 2.9, 2.08 and 1.68, respectively.

Due to the pH range of wines and tartaric acid solutions (generally at or lower than pH 3.7), Methyl orange (Sodium 4-{[4-(dimethylamino)phenyl]diazenyl}benzene-1-sulfonate) is selected as the pH indicator (its pH transition range is between 3 and 4). To prepare the methyl orange indicator, 0.1 g of methyl orange is dissolved in 80 mL of water. Then, ethanol (95%) is added to reach a total volume of 100 mL. The physical Physical properties of the used reagents are presented in Table 3.

#### 2.4.2. 3D Printed Support

**Figure 3:**
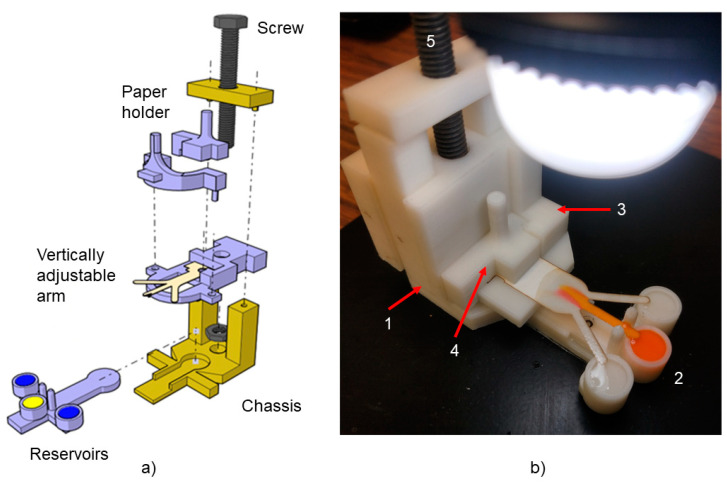
3D-printed support. (**a**) Assembly of the different parts. (**b**) Picture of a test under performance.

One of the challenges of the paper-based microfluidics diffusion-based sensors is achieving a concurrent contact between the inlets’ samples and the indicator. Therefore, a 3D-printed support has been designed to avoid the human error factor in substrate positioning and to achieve simultaneous flow between inlets. The 3D structure, as illustrated in Figure 3a,b, consists of 5 parts: the chassis, the reservoirs, the vertically adjustable arm, the paper holder and a screw.

The chassis (number 1 in Figure 3b) is a horizontal plate that stabilizes and bears the whole setup. The chassis has an internal thread (for the screw to enter in) and a section to hold and fix the reservoir.

To evaluate the effect of the inlets’ angle on the diffusion, three reservoir models are developed to fit the 30-degree, 45-degree and 90-degree paper strips (number 2 in Figure 3b). This part has three cylindrical containers, which are filled with 60 µl of the solution.

The other part is the vertically adjustable arm (number 3 in Figure 3b). This part is connected to the chassis using only the screw (number 5 in Figure 3b) and it enables the simultaneous contact between the reagents and the inlets of the paper strip. The paper holder (number 4 in Figure 3b) is fabricated to make sure that the paper is fixed and does not move during the tests.

The abovementioned parts are designed with CATIA V5 software and the resulting “.STL” files are printed by a ZORTRAX M200 3D -printer. The material used for fabricating the parts is Z-ULTRAT (ABS- Acrylonitrile Butadiene Styrene).

#### 2.4.3. Measurement Configuration

Since the diffusion width evolves while there is a species gradient (see Equation (3)), there is the need to establish a benchmark time to produce repeatable and accurate results. Once the diffusion width is established, this is measured through the RGB profile analysis of the reaction between the reagents and the pH identifier in the meeting zone. The measurement evaluates the intensity change in the green color channel. Initially, methyl orange has a solid yellow (light orange) color in its neutral form. When hydronium (H_3_O^+^) from the sample of interest (acids pH < 4) diffuse into methyl orange, the pH indicator experiences structural changes and reflects a red color. The beginning of the reaction causes a drop in the intensity of the green channel, since the yellow color shifts to red and subsequently the constituent green color index decreases.

To determine the measurement time, the moment when the intensity of the green channel starts to drop is set as the “Time zero” and the pictures for analysis are taken at certain intervals (60, 90, 120, 180 and 240 s) after the “Time zero”. Numerous trials showed that this strategy is legit, repeatable and can be applied to all the cases. The pictures of the measurement line (as shown in Figure 2c) are captured by the Dino-Lite MS325B microscope. The ambient and the projected light are the same in all the assays and the pictures are later analyzed by the ImageJ software.

Finally, the limit of detection value (*LOD*) is calculated through the following formula: (detailed description of the *LOD* is presented in the Appendix B.

(10) LOD=3.3×Sm

where *S* is the standard deviation between the actual values and the predicted values by the calibration plot and m is the slope of the regression line.

#### 2.4.4. Errors and Data Curing

There are two main sources of error in this work: numerical simulation errors and the empirical test errors. Careful efforts are made to guarantee that the mathematical model is in agreement with the true flow. The effect of discretization is considered and the cells’ sizes are chosen accurately to not influence the final results. Also, the residuals of the results between consecutive iterations are properly chosen to not produce a false convergence.

In the experimental section, the errors might have been caused by the staff and/or by the devices. Numerous experiments (at least 5 tests for every configuration of solutions on each model) are conducted in order to minimize these effects. Moreover each group of dataset is analyzed and the standard deviation (*S.D*) is calculated through:

(11) $$S.D=\sqrt{\frac{\sum_{i}^{N}{\left(C_{i}-\bar{C}\right)}^{2}}{N}}$$

Let $C_{i}$, $\bar{C}$, i and N be the value of interest at the ith pixel, mean value of interest in the population, arbitrary variable and the total number of population in the analysis, respectively.

In order to avoid the picture capturing errors (such as light reflection and the ambient light) the microscope is positioned in a manner to receive the minimum reflection and its position is kept still during the assays. Besides, the magnification rate is constant for all the models. Also, the ambient light is measured and the background light is adjusted to be uniform in all the tests.

Eventually, the Grubb’s test is applied to all the obtained results to detect the outliers at a 0.05 significance level to minimize the effect of the abovementioned errors.

## 3. Results and Discussion

### 3.1. Numerical Simulation

**Figure 4:**
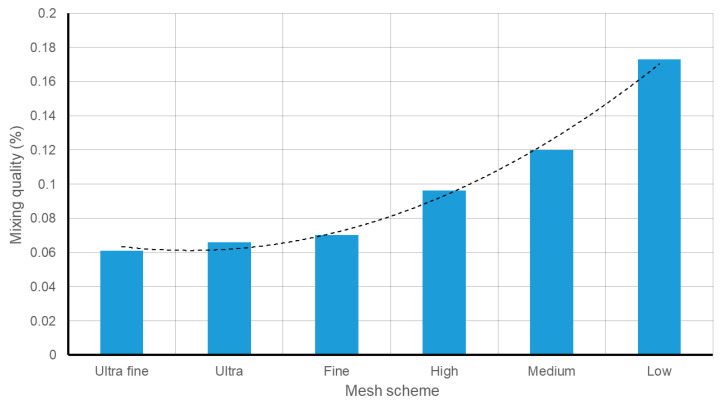
Mixing quality based on the applied mesh scheme.

A detailed report on the mesh refinement is presented in Appendix A: Grid study. The mixing quality is studied to see the effect of the mesh scheme on the produced result. Figure 4 shows a comparison of the different meshes. “Fine”, “Ultra” and “Ultra-fine” meshes provide similar results. For instance, the species distribution of the “Fine” mesh scheme only showed a 0.9% deviation from the “Ultra-fine” mesh design. Therefore, the “Fine” mesh distribution is selected to perform the study.

#### Numerical Analysis of Inlets’ Angle Effect on the Species Diffusion in the Porous Medium

Previous conventional microfluidics studies reported an enhancement of diffusion velocity, depending on the inlets’ angle [41]. Thus, four different substrate geometries with inlets’ angle of 30, 45, 60 and 90 degrees are modeled and simulated to study if the same effect is relevant in porous substrates.

**Figure 5:**
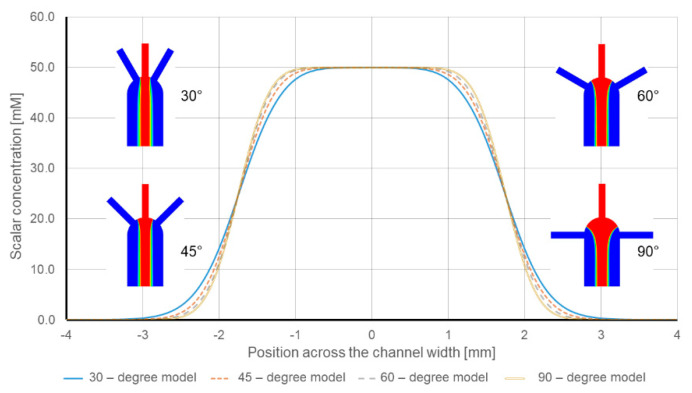
Numerical model results of the Species diffusion width with respect to inlets’ angle.

**Figure 6:**
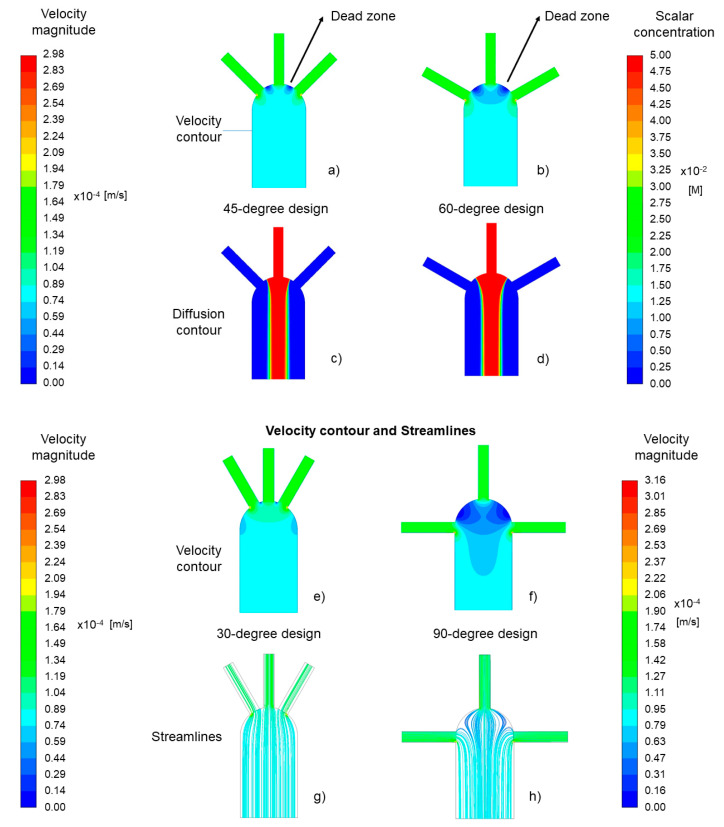
Velocity and diffusion contours of the 45-degree (**a**,**c**) and 60-degree designs (**b**,**d**). Velocity contours and streamlines of the 30-degree model (**e**,**g**) and the 90-degree model (**f**,**h**) are also illustrated.

Figure 5 shows the numerical simulation results of the diffusion width at the measurement line for the different models. According to the results, there is a reinforcing effect due to the inlets’ angle. When the lateral inlets are closer to the middle inlet (smaller angle), the diffusion width becomes wider. The reported diffusion width for the 90-degree model is 480 µm, while the species diffusion width is 550 µm in the 30-degree model. Figure 6 plots the flow lines for each model. In the case of the 90-degree model, the flow lines from lateral inlets enter the main channel perpendicular to the general direction of the flow. Since there is only one outlet in the design, the flow needs time and space to change direction. According to the results, this required time affects the diffusion. For instance, when the inlets’ angle reduces, the deviation from the general direction of the flow in the main channel decreases, and as a result, the species have more time and space to diffuse.

Figure 6 depicts the presence of regions (dead zones) between the inlets, where the flow velocity is at its minimum value. The comparison between the different models shows that these minimum velocity regions shrink by decreasing the inlets’ angle. Figure 6a,b shows that as the distance between the inlets increases, the developed dead zone becomes larger. The effect of these dead-zone areas on the diffusion width can be seen in Figure 6c,d. As shown in Figure 6, the diffusion width begins to grow in Figure 6c, where the angle is smaller compared to Figure 6d, which has a larger inlets’ angle.

Dead zones show an opposite relation with the diffusion width, a larger dead zone causes a narrower diffusion width at the measurement line. Therefore, the size and the location of the dead zones with diffusion contour can explain the differences of species distribution in different models.

### 3.2. Experimental Results

Several paper substrate geometries of the 30-degree, 45-degree and 90-degree models are prepared and tested to validate the numerical simulations results. To enhance accuracy, an appropriate substrate support is used for each model, and all the reservoirs are filled with 60 µL of solution.

#### 3.2.1. Relation between Inlets’ Angle and the Required Time for Measuring the Diffusion

The time required to develop a 1.5 mm of diffusion width in all the cases is analyzed to establish an accurate protocol to be measured with repeatability by ImageJ software. The evaluation of the diffusion width is conducted on the measurement line.

**Figure 7:**
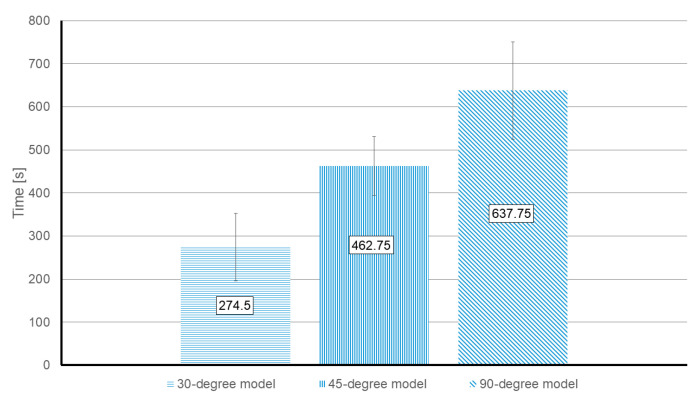
Experimental value of the required time to develop a 1.5 mm diffusion width at the measurement line. The reported values are the median time for each model by considering all the working reagents. The units are in seconds.

Figure 7 demonstrates that the required time is related to the inlets’ angle and increases from 274.5 s (±68.02 s) in the 30-degree model to 637 s (±113.3 s) in the 90-degree model. This is in agreement with previous numerical results.

**Figure 8:**
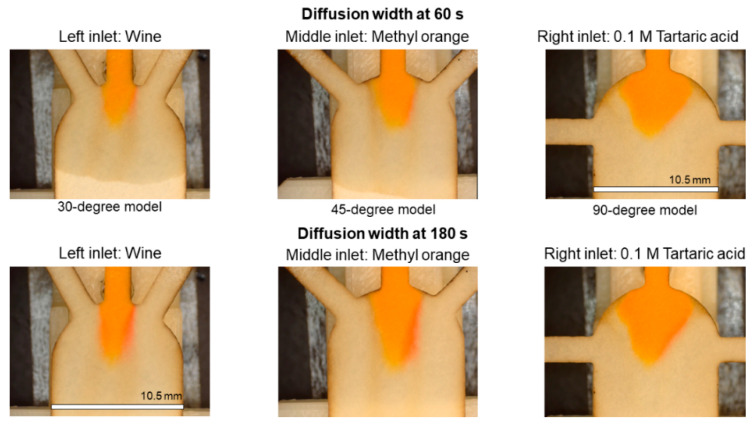
Diffusion widths at 60 s and 120 s after “Time zero” in paper strips with 30°, 45° and 90° of inlets’ angle. At all the cases, the wine is flowing through the left inlet and 0.1 M tartaric acid is entering through the right inlet. Scale = 10.5 mm.

Figure 8 compares the interaction between the pH indicator (middle inlet) and the reagents (0.1 M tartaric acid and wine) over the time, in the three different models (30-degree, 45-degree and 90-degree). In the measurements done at 120 s after the “Time zero”, the diffusion width increases considerably in the 30-degree and the 45-degree models. Moreover, in the 30-degree model, the shift of the pH indicator color is visible with the naked eye. Meanwhile, in the 90-degree model, the color change on the tartaric acid side is barely visually distinguishable.

Previous numerical results have shown that the dead zones appear in certain regions of the porous medium, and they modify the diffusion width at the steady-state. In agreement with the numerical results, the experimental outcomes reveal that the dead zones do also play a role during the transient flow. Therefore, the change in the inlets’ angle creates a different dead-zone area, which modifies the time taken for the flow to achieve a stable laminar flow.

#### 3.2.2. Effect of the Inlets’ Angle on the Diffusion Width

In order to define a protocol that produces repeatable and accurate results, the selected time to analyze the intensity of the green channel of the pictures is set to 240 s after the “Time zero”.

**Figure 9:**
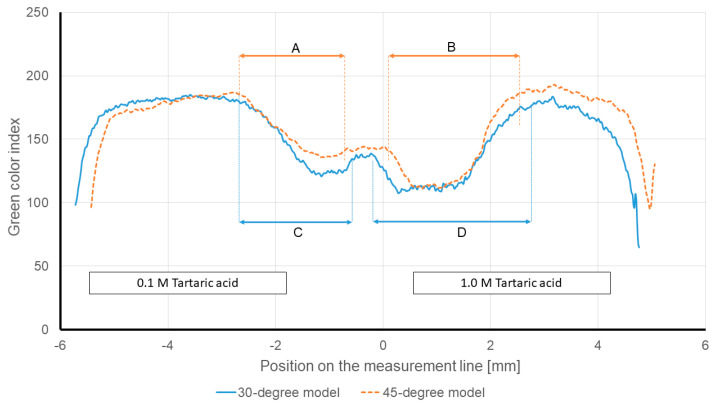
Intensity of the green color channel at the measurement line of different models at 240 s after the “Time zero”. In all the models, 0.1 and 1.0 M tartaric acid solutions were entered through the left and right inlets, respectively.

Figure 9 depicts the intensity of the green channel of the 30-degree and 45-degree models. In agreement with the numerical results, the diffusion width changes with the inlets’ angle. In both models, the same reagents (0.1 M tartaric acid from left and 1.0 M tartaric acid from right inlet) are introduced. The arrows A and C in Figure 9 show that both models (the 30 and 45-degree models) are able to reflect the presence of hydronium in the 0.1 M tartaric acid. As is expected, the 30-degree model is more sensitive than the 45-degree model, since the diffusion width is 1.89 mm for the same ionic strength compared to 1.825 mm (arrow C compared to arrow A).

In the same Figure, the arrows B and D show that both models can also reflect the presence of hydronium in the 1.0 M tartaric acid side (right side of the graph). Since the 1.0 M tartaric acid has a higher hydronium concentration compared to the 0.1 M tartaric acid, the intensity of the green channel analysis shows a wider diffusion width on the right side of the graph. The measured diffusion width for the 45-degree model is 2.164 mm (arrow B). While the diffusion width for the 30-degree model increases to 2.594 mm (arrow D). The mentioned results are not only in agreement with the provided justifications, but also, they show that the 30-degree model has higher sensitivity to the hydronium concentration.

**Figure 10:**
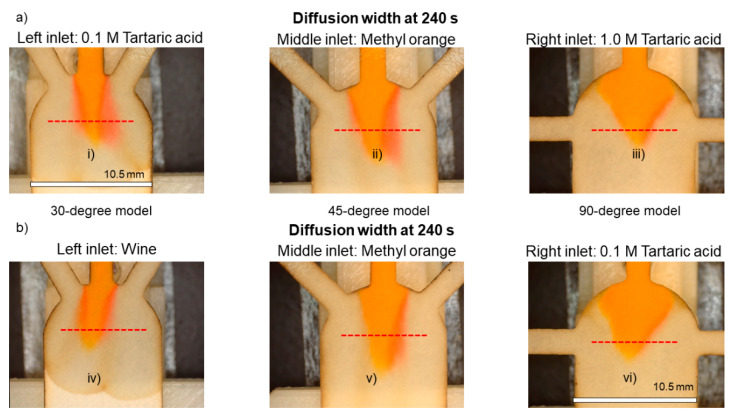
Visual comparison of diffusion development in different models. Measurement line is displayed as the dashed line.

Figure 10 plots a visual comparison of the diffusion development in the different models. In all the pictures in Figure 10a, the 0.1 M tartaric acid enters through the left inlet, and the 1.0 M tartaric acid is introduced via the right inlet. According to the pictures i and ii, the 30-degree and 45-degree models show a measurable response to the introduced reagents. On the other hand, in the pictures of Figure 10b, wine enters through the left inlet, and the 0.1 M tartaric acid is introduced via the right inlet. The comparison shows that only the 30-degree model (picture iv) displays a measurable response to the ionic strength of the wine. The 45-degree model requires further in-depth analysis. Meanwhile, the 90-degree model (iii and vi) is struggling to reflect the ionic strength even for the highest concentrations.

According to the results, if the ionic concentration decreases, the diffusion phenomenon would be more dependent on the geometrical characteristics of the substrate. Therefore, the most accurate geometry would be the 30-degree model. As predicted in the numerical models, the inlets’ angle modifies the way the solutions enter the main channel. Subsequently, the encounter of the reagents is affected. By taking into account the results and the provided justifications, it can be concluded that choosing a lower angle for the inlets facilitates the interactions between the fluids of different angles and improves the performance of paper diffusion-based sensors.

**Figure 11:**
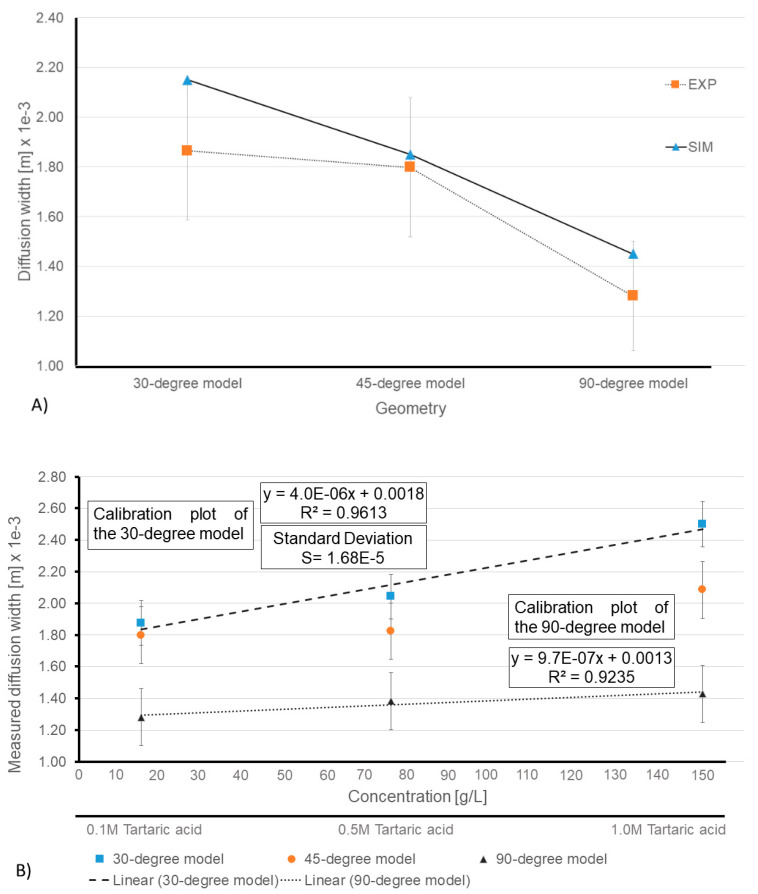
(**A**) Comparison between the diffusion widths in numerical results and the experiments. (**B**) Measured diffusion width based on the changes in the intensity of green channel and the calibration plots of the 30-degree and 90-degree models.

The results pointed out by the numerical model were validated experimentally. (Please see Figure 11A). According to the numerical results, the 30-degree model could provide a 2.15 mm of diffusion width. Meanwhile, the 45 and 90-degree models only provide 1.85 and 1.45 mm of diffusion width for the same ionic strength of the solution, respectively. On the other hand, regarding the experimental results, the diffusion width for the 30, 45 and 90-degree models were 1.865, 1.798 and 1.281 mm, respectively (detailed description of the reported diffusion widths of the experimental assays in mentioned in the next paragraph). By comparing the numerical and experimental results, it can be claimed once again that the experimental results support the numerical studies, and the numerical model has simulated the µPAD with acceptable accuracy.

Figure 11B displays the average reported diffusion widths in all the proposed models for the measured green color profile of the reagents (0.1, 0.5 and 1M tartaric acid). The *X*-axis is the concentration based on (g/L) unit, which was converted from the molar (M) unit (with respect to the molar mass of tartaric acid) and the *Y*-axis is the measured diffusion width based on the meter (m) unit, which was converted from the millimeter (mm) unit. Figure 11B clearly shows that the 30-degree model has a steeper regression line, and therefore, it is more sensitive to capture the differences between tartaric acid solutions. Meanwhile, the regression lines in the 45-degree and 90-degree are more gradual and demonstrate poor sensitivity. For instance, the 30-degree model displayed a 1.865 ± 0.28 mm and 2.478 ± 0.18 mm of diffusion width for the 0.1 M and 1.0 M tartaric acid reagents, respectively. However, the aforementioned values for the 45-degree model were 1.798 ± 0.28 mm and 2.085 ± 0.11 mm, showing only a 200 µm margin between different solutions. The reported margin for the 90-degree model was even worse, when the reported measurements dropped to 1.281 ± 0.22 mm and 1.429 ± 0.21 mm for 0.1 M and 1.0 M tartaric acid solutions, respectively. Moreover, based on the calculated regression line, the 30-degree model displayed a 13.9 g/L limit of detection, whereas the same value for the 90-degree model was reported as 34.6 g/L.

The results showed that, by choosing the inlets’ angle properly, even the lowest ionic concentration can be measured with the simplest detection techniques.

#### 3.2.3. Response Time Optimization for the 30-Degree Model

**Figure 12:**
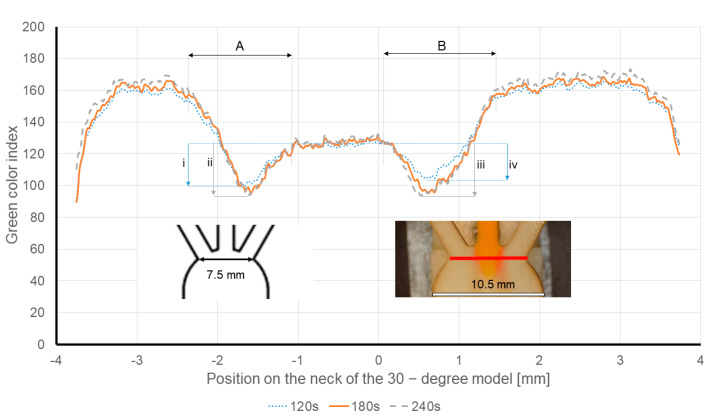
Intensity of the green channel of the diffusion on the measurement line at 120, 180 and 240 s after the time zero. Wine is injected through the left inlet and 0.1 M tartaric acid solution is entered via the right inlet. Red line shows the new measurement line at the neck of the 30-degree model. The dimensions are also illustrated in the pictures.

One of the major advantages of the µPADs is the possibility to provide results in a short turnaround time. Heretofore, the results showed that choosing a small angle can magnify the diffusion and shorten its required time. In order to optimize the proposed setup, the diffusion was analyzed at the inlets’ intersection of the 30-degree model. The new measurement line was chosen: first, to have the same evaluation position for all the cases, and second, to reduce the required time to evaluate the diffusion zone on the new measurement line (see Figure 12). The test configuration was similar to previous tests, except for the picture capturing time. Pictures were taken at 120, 180 and 240 s after the “Time zero”.

Figure 12 displays the overlap of the intensity of the green channels of the 30-degree model at 120, 180 and 240 s after the “Time zero”. In these experiments, the wine is soaked through the left inlet, and the 0.1M tartaric acid solution is entered via the right inlet. The red line plotted in Figure 12 shows the new measurement line at the neck of the 30-degree model. The arrows A and B show the diffusion width, and the arrows i–iv show the drop in the intensity of the green color channels.

As expected, in all the tests, the measured diffusion width (arrow A) remained constant and only showed insignificant changes over time. The measured diffusion width for the wine was 938.66 µm ± 14.05 µm, and apparently it was independent of time. For instance, the intensity of the green channel drop is 23.2 units for the wine 120 s after the “Time zero” (arrow i). While the same measurements at 180/240 s after the “Time zero” (arrow ii) only shows a drop of 25 units.

The evolution of the intensity of the green channel of the 0.1 M tartaric acid solution (arrow B) is also measured, being 975.33 µm ± 56.41 µm. However, the intensity of the green channel drop in the tartaric acid side seems to be more affected by the time compared to the wine samples. The drop in the intensity of the green channel is 32.48 units at 180 and 240 s (arrow iii) after the “Time zero”, while it is 23.16 units at 120 s. This means that the color change is intensified over time. Therefore, for pH measurements, longer times would be required to achieve accurate measurements in this type of sensor.

To measure the diffusion width, which is the physical value that correlates to the ionic strength, the evaluation at the new measurement line (neck of the 30-degree model) provides reliable results in 120 s and running the test for a longer time period does not alter the diffusion width, as it is plotted in Figure 12 (please see arrows A and B).

The obtained outcomes can be explained by the diffusion flux and the definition of porous medium. As it has been stated before, the smallest dead zones are reported in the 30-degree model. This can be translated into the fact that when the solutions from different inlets enter the main channel, they encounter each other, and they begin to interact (or diffuse) imminently. The existing hydronium from the wine and the tartaric acid solution diffuses into the pH indicator region. Since the hydronium concentration is limited, the resulting diffusion flow would be affected, and the diffusion width growth would be decelerated. Hence, it means that the hydronium ions diffuse further as the time passes, although at a lower rate. Meanwhile, it should be noted that the diffusion is occurring within a porous medium, which generates a flow perpendicular to the diffusion flow. While in the porous media there is a continuous flow from the inlet/s towards the outlet. It is true that the diffusion width increases over time, but there is also a bulk movement from the inlet to outlet. The diffusion is happening inside the porous medium and the hydronium advances further over time, but at the same time, the diffusion zone is moving towards the outlet due to the bulk movement. So, if the evaluation is carried out at a certain line, the measured diffusion width would be independent of the time, and it only relies on the initial flux, while the diffusion flow is affected by the hydronium concentration and time. Therefore, measuring the diffusion width over a line not only eliminates the time factor in the diffusion, but also provides the capability to compare the concentration of species in two solutions. Therefore, the proposed design is suitable for ionic strength measurements.

On the other hand, the drop in the intensity of the green channel is influenced by time. Especially, as the concentration of hydronium increases. The drop in the intensity of the green channel is due to the pH indicator’s color shift, and the color shift is caused by the transformation of the methyl orange molecule from the Azo structure to the Quinoid structure. The transform rate is highly affected by the amount of hydronium in the solution and results in the sedimentation of methyl orange salts. At lower hydronium concentrations the sedimentation is limited, but as the concentration increases the sedimentation is augmented dramatically and the salts even form fibers. As a result, the salts and fibers are stranded in the pores, and as the time passes, they accumulate and reflect a more intense color shift.

**Figure 13:**
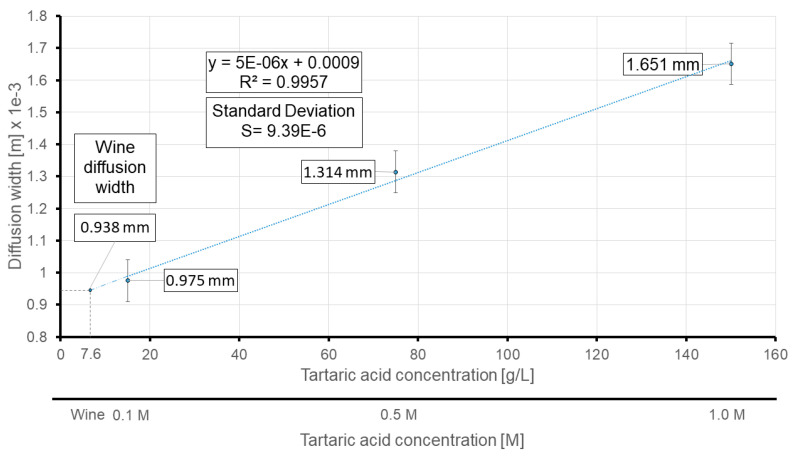
Calibration plot based on the average measured diffusion widths at the new measurement line (neck of the 30-degree model). The predicted concentration of wine is also displayed via extrapolation of the regression line. The units are in millimeters.

To validate the performance of the sensor, the diffusion width of the different solutions is measured. The wine shows an average diffusion width of 938.66 ± 14.05 µm, whereas the 0.1, 0.5 and 1.0 M tartaric acid solutions demonstrate an average diffusion width of 975.33 ± 56.41 µm, 1314.08 ± 69.06 µm and 1651.03 ± 34.84 µm, respectively. Figure 13 plots the calibration line obtained from the diffusion width at the inlets’ neck for different tartaric acid solutions.

Based on the obtained calibration plot, the proposed sensor predicts 7.6 g/L of total acid concentration for the wine sample. Alternatively, the literature HPLC measurements showed that the total acid concentration of white wines varies from 5.64 to 10.7 g/L [42]. The average acid concentration of the white wine, according to the literature, is 7.91 g/L, which is significantly in agreement with the predicted concentration of the wine under study by the sensor.

The limit of detection for the 30-degree model (at the inlets’ neck), according to the results, is equal to 6.2 g/L or 41.3 mM, which is an improvement compared to previous µPAD measurements. On the other hand, the obtained LOD legitimized the utilization of the proposed µPAD for ionic strength determination in wines, and once again showed that the diffusion widths of wine and 0.1M tartaric acid are in the same order and outcomes are in agreement with previous studies [43]. Although the reported LOD is not as remarkable as other techniques which require bulky measuring equipment, it should be noted that the proposed self-referencing sensor is designed and validated to be used in remote areas with minimum reliance on sophisticated and laborious detection and quantification techniques. Therefore, even being able to conduct quantitative measurements in less-developed regions can be considered as a great step forward.

On the other hand, the numerical and experimental results also revealed that the orientation of inlets is mainly affecting the neighboring inlets and the flow velocity. This can be translated into the fact that choosing appropriate inlets’ angle and type of substrate can expand the variety of tests to be performed. Therefore, the outcomes of this assay can be extrapolated to other studies and improve the µPADs’ designs.

Finally, it should be noted that in the experimental section of this assay, we used wine as a sample under analysis. Because in the wine production process, knowing the wine’s ionic strength can be later related to the capacity of the wine to be stored for longer periods. We selected the tartaric acid as a reference solution, since according to [42], the tartaric acid is the most abundant acid in wines. However, there are numerous other reagents that can be used for determining the ionic strength (for instance, 0.05M KCl and 0.25M NaOH (1%) solutions). Therefore, by choosing a different ionic strength reference, this sensor can be applied to numerous other applications.

## 4. Conclusions

This study proposes a diffusion paper-based sensor to take advantage of microfluidics, 3D fabrication methods and simple detection techniques to widen the capabilities of lab-on-paper platforms. Since the uncontrollable flow is the major drawback of the µPADs, a 3D-printed support was developed to guarantee the flow synchronization in the porous medium, and the flow was analyzed using CFD models.

A CFD model which took as reference the paper characteristics was developed and validated as a useful tool for the design of paper diffusion-based sensors. The model was used to evaluate the effect of the substrate geometry on the flow within the porous medium and to develop a sensor setup that could compare the ionic concentration of an unknown solution to a known solution quantitatively.

The experimental tests were in agreement with the numerical models and validated that less time was required to develop the diffusion zone if the inlets’ angles were smaller. For instance, the 30-degree model required 57% less time to develop a diffusion zone compared to the 90-degree model. Moreover, models with inlet angles bigger than 30° were unable to detect concentrations below 0.5 M.

Therefore, the 30-degree model was used to implement a paper diffusion-based sensor to evaluate the ionic strength of wines by comparison to different tartaric acid solutions. This novel sensor presents a limit of detection of 6.2 g/L and it is capable of evaluating the ionic concentration of commercial wines in 120 s without the need of any external equipment or trained personnel. Different substrates can be evaluated to further optimize the turnaround time of the results. This sensor strategy can be applied to other species and using the same model of different paper substrates can be evaluated to tune the generated capillary flow. As a result, the accuracy and the results turnaround time can be enhanced.

## Figures and Tables

**Table 1:** Paper substrate and cellulose fiber characteristics.

Property	Value
Density of Cellulose (ρ_cellulose_)	1.5 gr/cm^3^ [35]
Diameter of the cellulose fiber (d)	19.6 µm [35]
Average length of the cellulose fiber ($L_{f}$)	830 µm [35]
Density of Whatman grade 5 paper (ρ_W5_)	0.53 gr/cm^3^ [35]
Pore shape factor (φ)	140 [36]
Length of the substrate (L)	30 mm
Substrate main channel width (W_ch_)	10.5 mm
Substrate inlet channel width (w_i_)	2 mm
Substrate inlet channel length (l_i_)	15 mm

**Table 2:** Physical properties of fluids and physical characteristics of paper substrate.

Property	Value
Water density (at 25 °C)	998.2 kg/m^3^
Water viscosity (at 25 °C)	0.001003 kg/m·s
Diffusion coefficient of dye (D)	2 × 10^−10^ m^2^/s [37]
Porosity of the Whatman 5 porous media	0.6467
Viscous permeability	4.551 × 10^−15^ m^2^

**Table 3:** Physical properties of white wine and tartaric acid at room temperature (25 °C).

Property	Value
White wine density	1080 kg/m^3^
White wine viscosity	0.00148 kg/m.s [40]
Tartaric acid molar mass	150.078 g/mol
Tartaric acid viscosity	0.00121 kg/m.s (from producer’s catalogue)

**Figure A1:**
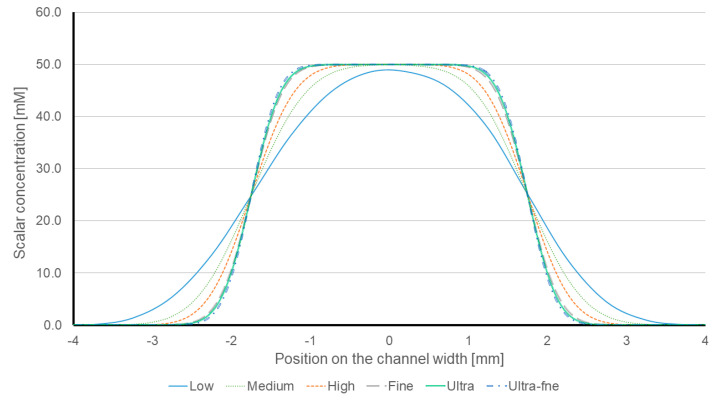
Distribution of scalar with respect to the mesh design in the 90-degree model.

## Appendix A. Grid Study

A mesh independency analysis ensures that the mesh density and distribution are not affecting the final results. We chose the 90-degree model as the basis for mesh refinement and the scalar diffusion as the variable of interest. Since the fluid was flowing through a porous medium, the flow velocity would be relatively small, and the viscous resistance would be considerably large. As a result, the mesh refinement did not have a significant effect on the velocity profile. Thus, we decided to monitor the scalar diffusion. Mesh refinement consisted of six grid groups. The minimum element size varied from 0.5 mm to 25 µm. The details of the studied grids are mentioned in Table A1.

**Table A1:** The details of the studied mesh distributions.

Grid	1 × 10^−4^ Element Size (m)	1 × 10^−5^ Minimum Surface Area (m^2^)	Minimum Orthogonal Quality	Number of Iterations
Low	5.0	25.1	0.833	88
Medium	2.5	11.68	0.787	110
High	1.25	6.01	0.661	165
Fine	0.5	1.93	0.69	317
Ultra	0.375	1.75	0.533	358
Ultra-fine	0.25	0.798	0.301	552

In all the cases, it was presumed that the flow velocity is 1.39 × 10^−4^ m/s. The fluid density and viscosity were set to 998.2 kg/m^3^ and 0.001 kg/(m.s), respectively. For mimicking the diffusion (species transport), a User-defined scalar was defined and dedicated to the main channel. The scalar’s concentration was set to 0.05 M and its diffusivity was calculated to be 1.29 × 10^−7^ kg/m.s (Please refer to Section 2.2.3 in this article).

Mixing quality investigations showed that the reported mixing qualities in the “Fine”, “Ultra” and “Ultra-fine” mesh refinements were quite similar. Whilst the “Low” mesh distribution showed a mixing quality of 17.3%, the reported mixing quality of the “Ultra-fine” scheme was 6.12%. Comparison between the mixing qualities of the “Fine” and “Ultra-fine” mesh designs only showed a 0.9% of difference. (Please see Figure 3 in the manuscript)

Moreover, according to the results (Figure A1), the “Fine”, “Ultra” and “Ultra-fine” mesh schemes provide the most similar results. Point to point comparisons showed that the “Low” mesh scheme has 58.6% of similarity to “Ultra-fine” mesh design. Meanwhile, the results of the “Fine” mesh distribution presented a 96.2% of similarity to the “Ultra-fine” mesh scheme.

Despite producing the most similar results, the number of required iterations for the results to converge was a determining parameter. Analysis revealed that the number of required iterations for the “Fine” mesh scheme was 42.6% less than the same value for the “Ultra-fine” mesh design and therefore, it was more efficient from the calculation-cost point of view. Considering all the aforementioned points, we decided to use the “Fine” mesh distribution for the numerical analysis.

## Appendix B. Limit of Detection

Limit of detection (LOD) expresses the minimum amount of a compound that can be detected in comparison to its absence. One of the most conventional methods for determining the LOD in experimental methods is by considering the generated standard deviation. In most of the assays the distribution of results can be illustrated by a regression line, expressed as:

(A1) y=mx+b

where *m* is the slope of the regression line and *b* is the interception point of the line with the *y*-axis.

By determining the regression line formula and obtaining the standard deviation (*S*) of the assays, the limit of detection can be found through:

(A2) $$LOD=3.3\times \frac{S}{m}$$
